# Recent advances in colonoscopy

**DOI:** 10.12688/f1000research.7567.1

**Published:** 2016-03-11

**Authors:** Thomas J.W. Lee, Shelley Nair, Iosif Beintaris, Matthew D. Rutter

**Affiliations:** 1 North Tyneside General Hospital, North Shields, UK; 2 Northern Region Endoscopy Group, North Shields, UK; 3 University Hospital of North Tees, Stockton on Tees, UK; 4 School of Medicine, Pharmacy and Health, Durham University, Stockton-on-Tees, UK

**Keywords:** Colonoscopy, colorectal cancer

## Abstract

Colonoscopy is an important and frequently performed procedure. It is effective in the prevention of colorectal cancer and is an important test in the investigation of many gastrointestinal symptoms. This review focuses on developments over the last 5 years that have led to changes in aspects of colonoscopy, including patient preparation, technical factors, therapeutic procedures, safety, and quality.

## Introduction

Colonoscopy remains the gold-standard investigation for inspecting the mucosa of the colon for pathology such as cancer, adenomas, or inflammation. It remains preferable, in many situations, to other imaging modalities such as computed tomography (CT) colonography or barium enema due to the capacity to intervene and sample or remove pathology encountered.

Emerging evidence over the last 5 years has led to important changes in the practice of colonoscopy throughout the patient journey. This review will focus on important practical developments and areas of interest. We will look at all aspects of colonoscopy, from the preparation, through the procedure and endoscopic therapy, to safety and quality assurance.

## Indications for colonoscopy

### Colorectal cancer screening

Colorectal cancer is the second most common cancer worldwide ^1^. There is variation in screening practices globally ^2^. Most screening programs employ a non-invasive test such as fecal occult blood testing to identify higher risk patients needing to undergo colonoscopy. Many screening programs are now using or moving towards using newer non-invasive tests, such as fecal immunochemical testing (FIT) or fecal DNA tests, which are associated with greater uptake and detection rates for adenomas and colorectal cancer ^3, 4^. In higher income settings, colonoscopy may be offered as the screening test. In the USA, a national survey estimated 65% of adults to be engaged with colorectal cancer screening, with colonoscopy being the most commonly used test ^5^. Such widespread colonoscopy screening appears to be reducing colorectal cancer incidence in the USA ^6^.

## Preparation for colonoscopy

An adequate level of bowel cleansing is critical for the efficacy of colonoscopy. Key quality indicators including cecal intubation rate and adenoma detection rate (ADR) are higher in patients with adequate bowel preparation ^7^. Furthermore, there is an improved rate of detection of flat lesions within the proximal colon in patients with adequate bowel preparation compared to those with inadequate preparation ^8^. Up to 1 in 5 colonoscopies are considered to have imperfect bowel preparation quality ^9^. Inadequate bowel preparation leads to lower ADRs and more missed lesions, and results in increased costs associated with rescheduling of the colonoscopy or organization of alternative investigations ^10^.

Recent years have seen the development of bowel cleansers that are more acceptable to patients. Preparations have evolved from large-volume (7–12 L) solutions with hypertonic saline to osmotically balanced solutions containing polyethylene glycol (PEG) and electrolytes. Introduction of split-dose bowel preparation regimens, where half the dose is given the day before the test and half on the day of the test, has significantly enhanced the ability to achieve high-quality cleansing with adequate preparation achieved in 85% compared with 63% in single-dose preparations ^11^. Split-dose regimens have resulted in improved ADRs and detection of flat lesions ^5^. The timing between the last dose of bowel preparation and colonoscopy has been shown to correlate with the quality of bowel preparation, and ideally should be less than 4 hours ^12^. Safety concerns have been raised regarding the use of sodium phosphate preparations and the associated risk of major fluid shift and electrolytes as well as chronic kidney disease from acute phosphate nephropathy ^13^. These risks make it an unsuitable first-line bowel preparation agent, and careful patient selection and appropriate cautions need to be taken before its use. In patients with renal failure, PEG is now the main recommended bowel preparation.

Patient education and willingness to participate in bowel preparation improves the outcome of cleansing. The delivery of both oral and written instructions for bowel preparation, as opposed to written alone, has been shown to be an independent predictor of adequate level of cleansing ^14^.

The above evolutionary changes in bowel preparation have increased its efficacy, safety, and patient tolerability, resulting in higher quality colonoscopy.

### Sedation

Current UK practice in colonoscopy is to use light conscious sedation or an unsedated approach. Maximum recommended doses of commonly used agents include midazolam (up to 5 mg) and fentanyl (up to 100 mcg) or pethidine (up to 50 mg). Unsedated colonoscopy, often using Entonox, is increasingly prevalent owing to improvements in technique. In the English Bowel Cancer Screening Program in 2014, 29.4% of colonoscopies were performed unsedated (personal correspondence, Professor M.D. Rutter). No difference in adenoma detection was observed in the unsedated group ^15^.

In some areas of the world, deeper sedation with propofol or general anesthesia is the preferred approach, although this may not be associated with improved adenoma detection, patient experience, or cost effectiveness, and has been shown to be associated with increased risk of complications ^16, 17^.

## Procedural developments

### Improving adenoma detection

An important objective of screening and surveillance colonoscopy is adenoma detection. Decreased incidence of post-colonoscopy colorectal cancer has been observed among colonoscopists with higher ADRs ^18^. An important paper by Corley *et al.* has also demonstrated an inverse relationship between ADR and interval cancer incidence and mortality from colorectal cancer. Based on 314,872 colonoscopies performed by 136 gastroenterologists, each 1.0% increase in ADR was associated with a 3.0% decrease in the risk of colorectal cancer (hazard ratio, 0.97; 95% confidence interval, 0.96–0.98) ^19^. The progress in understanding the importance and relevance of ADR has led to its recognition as the key performance indicator of colonoscopy and a useful tool for quality improvement, as discussed later in this paper.

A number of recent advances have sought to overcome some of the barriers to adenoma detection. Regular feedback of performance indicators to colonoscopists, ideally with peer comparators, has been shown to positively affect adenoma detection ^20^. The introduction of a simple training package and a bundle of simple maneuvers (withdrawal time >6 minutes, rectal retroversion, Buscopan use, and right lateral positioning on withdrawal) led to an increase in adenoma detection among a group of colonoscopists in the Quality Improvement in Colonoscopy (QIC) study ^21^.

New technologies to facilitate examination of difficult-to-access mucosa are currently being evaluated, although not all studies of quality improvement have been successful. These aim to increase the exposure of mucosa behind haustral folds, rectal valves, the ileocecal valve, and at flexures. Innovations included cap-assisted colonoscopy ^22^, the Third Eye® Retroscope® ^23^, wider-angle colonoscopes ^24^, and the Endocuff® device ^25^. Improving the definition of the colonoscopy image has been shown to increase adenoma detection, particularly small and flat lesions ^26^.

Chromoendoscopy is the technique of enhancing mucosal inspection using a dye applied to the mucosa. Commonly used dyes include indigo carmine and methylene blue. Dye spray chromoendoscopy has become the standard technique for colonoscopic surveillance for dysplasia in inflammatory bowel disease, increasing pathology detection and reducing unnecessary biopsies when compared to random biopsy protocols ^27^.

Dye spray colonoscopy may also increase adenoma detection in routine colonoscopy, but the incremental gain in detection of diminutive lesions is offset by increased procedure time ^28^.

Electronic image processing allows “virtual chromoendoscopy” with mucosal enhancement without physical dye application. Systems such as Narrow Band Imaging (Olympus), FICE (Fujinon), and i-scan (Pentax) have made such technology widely available. These techniques are particularly useful for lesion classification but have not consistently been shown to improve adenoma detection. The NICE system and Kudo’s pit pattern can help differentiate hyperplastic lesions from neoplastic lesions and identify higher grades of dysplasia that may be associated with submucosal invasion. This has become an important tool in planning management of lesions and deciding whether a lesion is resectable endoscopically or may require a surgical approach.

Improved lesion interrogation using electronic image enhancement has been proposed as an alternative to conventional polypectomy and histology. The American Society of Gastrointestinal Endoscopy (ASGE) has proposed a Preservation and Integration of Valuable endoscopic Innovations (PIVI) statement identifying criteria that a resect and discard strategy would have to meet to be acceptable for widespread use. An important driver for this strategy is the cost associated with pathological examination of small polyps which have an extremely low risk of containing advanced dysplasia. The DISCARD study demonstrated that in a single center with expert endoscopists, a resect and discard strategy accurately recognized adenomas with a sensitivity of 94% and accurately predicted appropriate surveillance strategy in 98% ^29^.

## Water-assisted colonoscopy

The first report of a water-based colonoscopy technique was from Falchuk and colleagues in 1984, which showed that water infusion facilitates scope insertion in patients with diverticulosis ^30^. Water-assisted colonoscopy (WAC) involves water infusion during scope insertion, instead of traditional air or CO _2_ insufflation. Main variations include water immersion (WI), during which water is infused to inflate the lumen during scope insertion and then aspirated during withdrawal, and water exchange (WE), where removal of infused water occurs predominantly during insertion ^31^.

Recent reports have shown that WAC, especially the WE technique, may lead to improved patient comfort with less sedation, aid in completion of difficult or previously incomplete procedures (due to angulations or redundant colons), as well as increase ADRs, the latter being a well-acknowledged predictor of interval cancer risk between colonoscopies ^14, 32, 33^. Therapeutic indications of WAC have also been described, such as endoscopic resolution of sigmoid volvulus in patients with high surgical risks, as well as polypectomy ^34^. Underwater endoscopic mucosal resection (EMR) has recently been proposed as an option in the excision of challenging lesions, such as in cases of failed conventional EMR and recurrent polyps, with promising outcomes in terms of recurrence and complication rates ^17, 35^.

Prolongation of cecal intubation time (CIT) is the main limitation of WAC. Some studies point to a significant prolongation, while others describe similar CIT ^36, 37^. With regard to safety, data so far show that the procedure is safe and does not interfere with patients’ fluid and electrolyte status ^36^.

## Management of colonic pathology

### Management of large colonic polyps

Endoscopic removal of large pre-malignant or suspected early malignant gastrointestinal (GI) lesions poses a challenge in that a complete resection that allows for staging and appropriate further management is warranted in these cases.

Traditionally, EMR, a technique that comprises submucosal injection of lifting solution followed by snare excision, has been used for such lesions. However, EMR is considered suboptimal for large lesions where there is an increased risk of submucosal invasion and therefore a need for a more oncologically sound, en-bloc resection that allows adequate staging. With piecemeal EMR, recurrence rates are significant (16% in a large Australian series), but recurrence is manageable endoscopically in the majority of cases ^38^ for colorectal lesions larger than 10 mm ^39^. The obvious need for a technique that allows for en-bloc removal of large, advanced esophagogastric and colorectal lesions recently led Japanese endoscopists to develop endoscopic submucosal dissection (ESD) as a more efficient alternative. This technique is based on the use of endoscopic knives to achieve a deep submucosal, en-bloc excision that allows for accurate histopathologic staging ^40^. Excellent pre-excision lesion assessment is warranted based on size, morphology, and surface pattern. To justify an attempt of endoscopic resection, advanced imaging modalities, mainly high-definition and chromoendoscopy, are utilized to achieve a meticulous lesion assessment.

Therapeutic indications of ESD in the colorectum include polyps that are suspicious of early neoplastic submucosal involvement as defined by endoscopic appearance (lesions with focal depression, irregular surface patterns, or poor submucosal lift), polyps larger than 2 cm, and polyps that pose difficulties to conventional EMR, such as areas of recurrence after previous polypectomy ^23, 34^.

Accurate recognition of colonic lesions with submucosal invasion can be challenging, and considerable variation in proficiency has been demonstrated among western endoscopists ^41^.

It should be stressed that ESD is a highly demanding modality in terms of endoscopist skill and requires specialized training and a certain volume of procedures to ensure competency ^42^. Perforation is a potentially severe complication of the technique, with a risk of 6–20% in colorectal lesions ^43, 44^. Surgery is still considered the gold standard for the treatment of most lesions that also fulfill criteria for ESD, although the latter appears to be superior in terms of periprocedural morbidity and mortality when performed by experienced endoscopists ^23, 34^.

Discussion of the management options for large colonic polyps by a multidisciplinary team is recommended, as regional variation in surgical management rates has been observed in England ^45^. Recent consensus guidelines on the management of large non-pedunculated colonic polyps have suggested a range of key performance indicators that aim to measure and improve standards of management of large polyps ^46^. There is likely to be a variation in performance of advanced polypectomy techniques and a subsequent variation in recurrence rates (which may be associated with risk of interval cancer) ^47^.

### Serrated lesions

Previously, the majority of colorectal cancers were thought to arise from the “adenoma-carcinoma” sequence as a result of an accumulation of genetic mutations ^48^. This process is slow, taking 10–15 years for normal mucosa to progress to malignancy, thus offering the opportunity of a long latent phase in which to detect and remove the malignant precursor (adenoma).

Recent years have seen the increasing recognition and understanding of an alternative pathway for the development of colorectal cancer. This arose from the appreciation of the hyperplastic polyposis syndrome, in which multiple hyperplastic polyps are present in the colon and associated with an increased lifetime risk of colorectal cancer of 20–50% ^49^.

Serrated lesions are now thought to represent an alternative pathway to colorectal cancer through mutations in *BRAF*, CpG island methylation, and subsequently methylation of *MLH1* ^50^. Hyperplastic polyps, which are very common and occur in up to 95% of individuals, progress to sessile serrated adenomas (SSAs), of which a proportion may have cytological dysplasia. A separate, morphologically and histologically different polyp called a traditional serrated adenoma (TSA) is recognized. SSAs or TSAs can be challenging to detect at colonoscopy due to their flat appearance and relative lack of differentiation from the background mucosa. They tend to occur in the proximal colon where lesion detection is impaired by prominent haustral folds ^51^.

Serrated lesions are thought to be clinically important due to their association with the presence of synchronous advanced neoplasia ^52^. They may also be a significant cause of advanced neoplasia or cancer detected following colonoscopy, presumably due to the increased likelihood of not being detected and subsequent rapid progression ^53^.

Increased awareness of serrated lesions and appreciation of their subtle appearance may aid detection ^54^. They commonly have an adherent mucosal cap, which obscures detection. Recently, an open shape pit pattern (type II-O) has been described to aid differentiation of dysplastic SSA from hyperplastic polyps; however, the clinical applicability of this is limited ^55^.

## Post procedure management

### Complications

Recognized important or commonly occurring complications of colonoscopy include bleeding, colonic perforation, and those related to sedation or anesthetic use. Current estimates of the frequency of such events depend on the indication for the colonoscopy, relevant patient factors, such as comorbidity, and whether endoscopic therapy is delivered during the procedure.

In the English NHS Bowel Cancer Screening Program, the overall rate of bleeding following colonoscopy is 0.65%, bleeding requiring transfusion 0.04%, and colonic perforation 0.06%. Risk of adverse events increases if polypectomy is performed. Factors relating to the polyp, including cecal location and increasing size, are associated with increasing risk of bleeding or perforation ^56^.

Endoscopic management of bleeding and perforation, including clip placement, over-the-scope closure devices, and endoscopic suturing techniques, may reduce the need for surgical intervention following such complications ^57, 58^.

## Quality assessment

Measurement and monitoring of colonoscopy quality is central to ensuring consistently high levels of performance, patient experience and safety. A variety of key performance indicators are used to measure colonoscopic performance, the most widely used being ADR. This requires histological confirmation of polyp type and is therefore potentially time consuming. Polyp detection rate is simpler to measure and can be used as a surrogate marker ^59^. The importance of ADR as a measure of colonoscopic performance is discussed earlier in this review.

Limitations of ADR include its restriction to counting only one or more adenomas. Detection of multiple adenomas is not reflected. In patient populations with a high prevalence of adenomas, such as higher risk screening populations, measures of the total number of adenomas detected, such as mean number of adenomas per procedure, may be more appropriate ^13^.

Other technical measures of colonoscopic performance, including cecal intubation rate, complication rates, and sedation rates, are widely used. Patient-reported measures are under-represented, and a validated patient-reported experience measure for colonoscopy could improve our ability to measure and enhance patient experience.

The ability to benchmark individual or unit measures of colonoscopic performance against regional or national measures is crucial to continuous improvement in quality. The National Endoscopy Database (NED) Project in the United Kingdom and the GI Quality Improvement Consortium (GIQUiC) initiative in the USA aim to provide high-quality, large-volume data to generate individual, regional, and national metrics to aid benchmarking, drive quality improvement, and identify gaps in quality or understanding that could be improved through further research.

## Conclusion

Colonoscopy is a commonly performed investigation. In the era of mass population screening for colorectal cancer, it is being performed more frequently than ever before. Advances in patient preparation, technical components of the procedure, and management of pathology will contribute to improvements in performance quality, safety, and ultimately patient experience.
